# Mobile DNAs and switching mating types in yeast

**DOI:** 10.7554/eLife.58403

**Published:** 2020-06-09

**Authors:** Laura N Rusche

**Affiliations:** Department of Biological Science, University of Buffalo, The State University of New YorkNew YorkUnited States

**Keywords:** homing endonucleases, inteins, mating-type switching, *Torulaspora*, *Lachancea*, yeast, *S. cerevisiae*, Other

## Abstract

The gene that allows budding yeast cells to switch their mating type evolved from a newly discovered family of genes named weird *HO*.

**Related research article** Coughlan AY, Lombardi L, Braun-Galleani S, Martos AAR, Galeote V, Bigey F, Dequin S, Byrne KP, Wolfe KH. 2020. The yeast mating-type switching endonuclease HO is a domesticated member of an unorthodox homing genetic element family. *eLife* **9**:e55336. doi: 10.7554/eLife.55336

Biologists working in evolution are often surprised when they find a ‘missing link’ with features that could not have been predicted from the features of its predecessors and successors. Such findings demonstrate the wonderful meandering path that evolution can take, with fitness rather than simplicity determining the route. One recent example is the discovery of a missing link in the evolutionary trajectory of a gene called *HO* that is involved in mating in *Saccharomyces cerevisiae* and some other species of budding yeast.

Individual yeast cells in these species belong to one of two mating types, known as ‘a’ and ‘α’, and mating occurs when a cell of one type fuses with a cell of the other type. However, if a cell is isolated from potential mating partners, it can 'switch' to the other type and self-mate with its own daughter cell. Switching is triggered by the *HO* gene, which encodes an endonuclease that specifically cuts the genomic locus (called *MAT*) that determines the mating type of the cell (Lee and Haber, 2015). This cut is repaired by copying a silent copy of the gene for the other mating type and inserting it into the *MAT* locus.

Switching between mating types is thought to have evolved in two stages (Hanson and Wolfe, 2017). First, the three loci that carry the mating-type genes (the active *MAT* locus and the two loci with the silent genes) came together in one genome through gene duplications and genome rearrangements. Although this allowed switching to occur, the process was inefficient. Second, the *HO* gene emerged and increased the frequency of switching. However, the evolutionary history of this gene is still unclear (Haber and Wolfe, 2005; Keeling and Roger, 1995).

It has been suggested that the *HO* gene evolved from mobile DNA elements called homing inteins, which ‘home’ into the coding sequence of a specific host gene. Specifically, a homing intein in yeast called *VDE* has a similar sequence to *HO* and is thought to be the evolutionary ancestor of this gene. The protein encoded by the *VDE* gene is initially expressed within its host protein; it then extracts itself and goes on to cut copies of the host gene that lack the intein sequence. This cut triggers a repair reaction similar to the one induced by the endonuclease encoded by *HO*, and results in *VDE* being copied from an occupied gene to an empty host gene. Thus, both *VDE* and *HO* code for proteins that initiate DNA repair reactions that change the genetic sequence of a cell. However, there are differences: *HO* is free-standing and does not self-splice, whereas *VDE* is embedded in a host gene; moreover, the protein produced by *HO* also contains a zinc finger domain that binds to DNA.

Now, in eLife, Kenneth Wolfe and co-workers from University College Dublin and Université Montpellier – including Aisling Coughlan as first author – report the discovery of a gene family which represents a missing link between *VDE* and *HO* (Coughlan et al., 2020). Similar to *HO*, the genes in the new family – named *WHO* for weird *HO* – have an intein-like cutting domain fused to a zinc finger domain (Figure 1A). Coughlan et al. discovered *WHO* genes in the genomes of yeast species that belong to the *Torulaspora* and *Lachancea* genera, and found them clustered next to a gene called *FBA1*. This arrangement suggests that *WHO* genes are mobile DNA elements that have inserted themselves at the end of the *FBA1* gene. Furthermore, fragments of *FBA1* can be found interspersed among the *WHO* genes, suggesting that *FBA1* is cut and partly duplicated during insertion.

**Figure 1.**
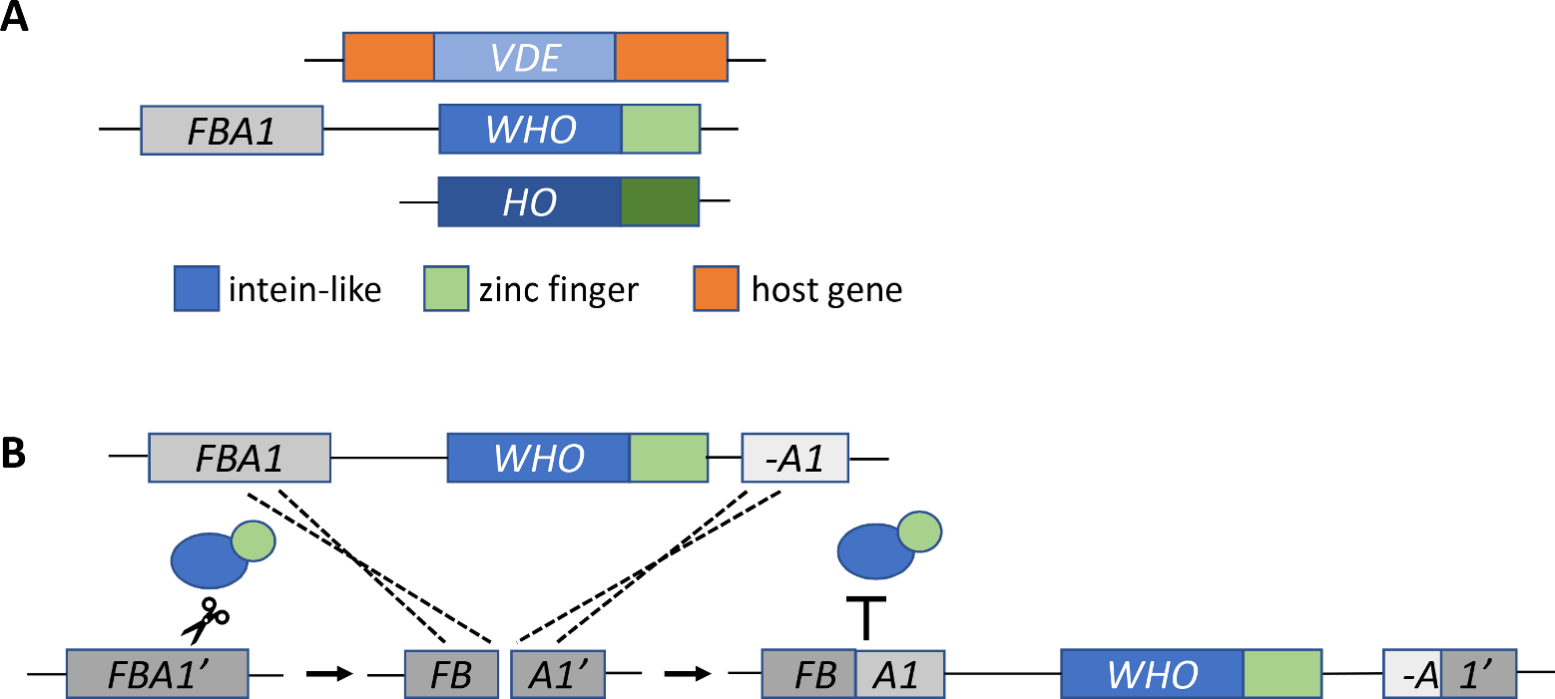
The missing link in the evolution of *HO*. (**A**) The *HO* gene is structurally similar to two other mobile DNA elements, *VDE* and *WHO*. All three genes have an intein-like domain (blue rectangle) that includes an endonuclease domain: in *HO* this domain cuts DNA at the *MAT* locus. The *WHO* and *HO* genes also have a zinc finger domain (green). The *WHO* gene is also located next to its target, the *FBA1* gene. (**B**) Coughlan et al. propose that the *WHO* gene homes into the *FBA1* gene as follows. First, the enzyme encoded by the *WHO* gene (blue and green ovals) cuts a target gene, represented as *FBA1’* (dark gray box), which is not flanked by *WHO*. Next, the break is repaired using a template of the *FBA1* gene (medium gray) which is adjacent to the *WHO* gene and a fragment of *FBA1* (A1, light gray). The template sequence (including the *WHO* gene) is copied and inserted into the cleaved site of the target gene. Recombination between the target *FBA1’* gene and the template *FBA1* gene creates a new *FBA1* gene (dark and medium gray) and *FBA1* fragment (light gray and dark gray), which sit on either side of the *WHO* gene. The newly recombined *FBA1* gene is no longer susceptible to cleavage by the enzyme encoded by *WHO* (represented by the T symbol).

To find out whether the cutting domain in the *WHO* family of genes targets *FBA1*, Coughlan et al. studied a gene called *WHO6* that comes from the species *Torulaspora delbrueckii.* The experiments were carried out in *S. cerevisiae* cells that contained a copy of the *FBA1* gene from *T. delbrueckii*. If the enzyme encoded by *WHO6* cuts the *TdFBA1* target gene, cell division will be halted until the DNA is repaired. Coughlan et al. found that when *WHO6* was overexpressed, cells that contained the *TdFBA1* gene were less likely to survive. The only cells that survived were those in which the *TdFBA1* gene had been modified to make it resistant to further cutting (Figure 1B).

These results indicate that the *WHO6* gene can trigger a repair reaction at the site of *FBA1*. Presumably, if the *FBA1* sequence used to repair the damage is adjacent to a *WHO* gene, this would allow *WHO* to home into *FBA1* (Figure 1B). This homing mechanism is surprisingly different from that of *VDE*, highlighting the meandering path of *HO* evolution. Moreover, it suggests that the structural features of *HO,* such as the zinc finger, were not originally selected for switching mating types.

The discovery that a *WHO*-like gene is a stepping stone on the evolutionary path to the *HO* gene raises a number of questions. For example, how did the preferred cut site shift from *FBA1* to *MAT*? The previous hypothesis was that acquiring the zinc finger domain was connected to the specificity for *MAT* (Haber and Wolfe, 2005). However, *WHO* genes do not cut *MAT* despite having the zinc finger domain. An answer may lie in the repeated homing of *WHO* genes into the same *FBA1* locus, which requires each incoming *WHO* gene to have a slightly different cutting specificity. Perhaps as new *WHO* variants were selected, one also happened to cut *MAT*. In this regard, it would be interesting to determine how the amino acid sequence of the proteins coded by *WHO* genes impacts the specificity of the cut site.

It is puzzling that the proteins encoded by *VDE* and *HO* are active in different cell types (haploid versus diploid), and that their activities are regulated in different ways: *HO* is only transcribed at certain times, whereas *VDE* is always transcribed but only localized to the nucleus at certain times (Gimble and Thorner, 1992; Nagai et al., 2003; Bakhrat et al., 2006; Stillman, 2013). How did these changes take place? An intriguing possibility is that the acquisition of the zinc finger domain, which contains a nuclear localization signal, was a key step in the regulation of the *HO* gene diverging from *VDE*. Thus, how the zinc finger domain contributed to the evolution of *HO* may have been different than originally imagined.

Another yeast species called *Kluyveromyces lactis* lacks the *HO* gene and instead initiates mating-type switching using two other genes that evolved from different DNA mobile elements (Barsoum et al., 2010; Rajaei et al., 2014). This raises the question of whether mobile DNA elements benefit from triggering the switch between mating type. Alternatively, they may have been captured by yeast because of their ability to re-arrange DNA, which makes them well-suited for switching.

At first glance, switching seems unlikely to benefit a homing element such as *WHO,* as it allows haploid cells to self-mate, preventing the introduction of new versions of a gene into which the element can home. However, generating a diploid cell does allow the yeast to produce spores which promote the long-term survival of the organism. The benefit of mating-type switching is further supported by the fact that this process has evolved eleven independent times in budding yeast (Krassowski et al., 2019). Perhaps, by promoting the survival of the host cell, the homing element preserves itself.
